# Editorial: Advances in alternative crop production and valorization in salt-affected areas

**DOI:** 10.3389/fpls.2024.1384030

**Published:** 2024-02-27

**Authors:** Abdelaziz Hirich, Atul Bhargava

**Affiliations:** ^1^ Agriculure in Marginal Environment Program, African Sustainable Agriculture Research Institute (ASARI), Mohammed VI Polytechnic University (UM6P), Laayoune, Morocco; ^2^ Department of Botany, Mahatma Gandhi Central University, Bihar, India

**Keywords:** quinoa, irrigation, salinity, forage crop, yield

## Introduction

Salinity is a major problem affecting agricultural activity in many regions of the world (Bhargava and Srivastava, 2020; FAO, 2021). Biosaline agriculture and crop diversification through the introduction of alternative crops are key solutions to overcome this problem and better valorize salt-affected lands. Traditional crops face many challenges caused by abiotic and biotic stresses, including salinity, drought, pests, and disease (Bhargava and Srivastava, 2013; Dubey et al., 2020). Alternative crops that can replace common crops in specific geographical areas can be quite beneficial in achieving specialized benefits in the farming system (Elouafi et al., 2020). In addition, the high value products of alternative crops have the potential to greatly improve the income of farmers. With increasing salinity affecting arable land globally, scientists are making great efforts to introduce, evaluate and improve new and alternative crop strains. However, more research is needed to screen for high yielding varieties, understand tolerance mechanisms, optimize cropping practices, and develop value chains to ensure the success of alternative crops in marginal environments.

In this special Research Topic of Frontiers in Plant Science, the articles concentrated on four main themes: (1) physiological and biochemical responses of alternative plants to salinity stress, (2) growth and agronomic performance of alternative crops under saline conditions, (3) role of organic amendment and fertilization to mitigate adverse effects of salinity on crop and (4) role of alternative crop as a source of quality food and feed.

## Physiological and biochemical responses of alternative crops to salinity stress

Plants deploy several strategies to cope with salinity stress at almost all levels to survive and thrive in salt affected soils. For example, quinoa, a well know salt tolerant crop, uses several mechanisms including osmotic adjustment by accumulating cheap osmolytes such as sodium and their compartmentalization in the vacuoles to avoid its interference with the potassium in the cytosol (Hirich et al., 2014). It was also shown that quinoa has the ability to accumulate more complex compounds such as proline and metabolites such as saponins and polyphenols under increased salinity to resist salt stress (El Mouttaqi et al.). Salinity stress can also induce oxidative stress in plants which can cause damage to cellular components. El Mouttaqi et al. also demonstrated that quinoa antioxidant activity increased under high salinity for neutralizing the reactive oxygen species (ROS) and preventing their interference with regular metabolism. Blue panicgrass (*Panicum antidotale* Retz.), another salt tolerant alternative crop, uses a strategy known as salt exclusion (in reference to sodium) as shown by (El Mouttaqi et al.). In fact, blue panicgrass subjected to salinity stress maintains a high K^+^/Na^+^ ratio in the shoots and a low ratio in the roots with higher Na content in roots and higher K content in the shoots.

## Growth and agronomic performance of alternative crops under saline conditions

Alternative crops, contrary to the conventional crops, are able to grow and produce satisfactory yield even in salt affected lands (Hirich et al., 2021). Sometimes, even traditional landraces and cultivars of conventional crops exhibit this potential. For example, it has been revealed that traditional tomato varieties improve fruit quality without a notable effect on fruit yield under moderate salt stress (Egea et al.). Furthermore, it was noticed that underexploited and alternative crops such as quinoa and amaranth had great potential to improve degraded soils of the Spanish Southeast area, due to their agronomic rusticity in marginal environments. El Mouttaqi et al. found that increased salinity levels resulted in a reduction of most agro-morphological parameters, but did not significantly affect root development. The same study showed that although salinity affected seed yield in quinoa, appreciable yield (0.7 t/ha on average) was obtained even under conditions of extreme water salinity (20 dS/m). In another study, El Mouttaqi et al. showed that blue panicgrass produced 74 t/ha of fresh forage biomass under irrigation in high salinity levels (16.3 dS/m) which was comparable to traditional forages grown under freshwater conditions (Iptas and Acar, 2006). Sesbania pea is another example of alternative crops, highly recommended for saline soils especially due to its ability to fix atmospheric nitrogen. Zhu et al. reported that sesbania pea grown in saline soil (EC= 10.87 mS/cm) could produce ample fresh biomass reaching up to 50 t/ha after 163 days of growth.

## Role of organic amendment and fertilization to mitigate adverse effects of salinity on crop

Excessive salts in the soil can lead to osmotic stress, ion toxicity, and nutrient imbalances, which negatively impacts plant health (Okon, 2019). The use of organic amendments and fertilization can play a crucial role in mitigating the adverse effects of salinity on crops by improving soil structure, increasing nutrient availability, enhancing microbial activity and reducing sodium toxicity. El Mouttaqi et al. demonstrated that organic amendment application under salinity conditions positively enhanced quinoa growth and productivity regardless of the amendments type. Furthermore, organic amendments improved seed quality, especially the saponin content that was decreased due to the application of organic amendments which is an important finding in view of the fact that saponin accumulation in the seeds is a limiting factor for quinoa transformation and valorization. Regarding fertilization, Zhu et al. showed that the application of 360 kg·hm^-2^ of nitrogen and 180 kg·hm^-2^ of phosphorus fertilizer has significantly improved growth and biomass yield with enhanced antioxidant capacity of sesbania pea in saline soils.

## Role of alternative crops as a source of quality food and feed

Alternative crops can play a significant role in providing sustainable source of good quality food and feed, offering various benefits as compared to traditional crops, thanks to their rusticity and resistance to adverse biotic and abiotic stresses (Elouafi et al., 2020). Salinity levels are known to increase lycopene, lutein, β-carotene, and violaxanthin levels through increased carotenoid-related gene expression, and carotenoid biosynthesis in tomato (Leiva-Ampuero et al., 2020). Likewise, salinity has been reported to induce accumulation of flavonoids, flavonol, phenolics and terpenes in plant systems (Arif et al., 2020; Dubey et al., 2021). Egea et al. highlighted the role of salinity stress in improving fruit and seed quality, especially by increasing secondary metabolite accumulation in the fruits, which are very important as nutraceuticals for the human diet. For instance, in Spain, much attention goes to traditional landraces of tomato especially by smallholder’s farmers due to their adaptation to the mediterranean environment constraints and also their improved fruit quality under salinity stress and other abiotic stress growth conditions. Egea et al explored the opportunities of exploiting natural variation of halophytes, particularly in pseudocereals like quinoa and amaranth. El Mouttaqi et al. demonstrated that salinity resulted in increased phenolic content and antioxidant activity in quinoa seeds which clearly indicated that salinity had beneficial impact in terms of improving food quality. Regarding feed, it was demonstrated that blue panic grass irrigated with highly saline irrigation water produced a high-quality feed (El Mouttaqi et al.). In fact, protein content reached 17 and 22% under 20- and 40 days cutting time intervals, respectively which was comparable to alfalfa irrigated with fresh water (Ferreira et al., 2015).

Thus, soil salinity is of prime concern since it alters various physiological, biochemical, and molecular functions of plant systems.

## Author contributions

AH: Writing – original draft, Writing – review & editing. AB: Writing – original draft, Writing – review & editing.
